# Cellular Senescence: Mechanisms and Therapeutic Potential

**DOI:** 10.3390/biomedicines9121769

**Published:** 2021-11-25

**Authors:** Zehuan Liao, Han Lin Yeo, Siaw Wen Wong, Yan Zhao

**Affiliations:** 1 School of Biological Sciences, Nanyang Technological University, 60 Nanyang Drive, Singapore 637551, Singapore; b180044@e.ntu.edu.sg; 2 Department of Microbiology, Tumor and Cell Biology (MTC), Karolinska Institutet, Biomedicum, Solnavägen 9, SE-17177 Stockholm, Sweden; 3 School of Chemical and Biomedical Engineering, Nanyang Technological University, 62 Nanyang Drive, Singapore 637459, Singapore; b170004@e.ntu.edu.sg

**Keywords:** senescence, cancer, aging, senotherapy

## Abstract

Cellular senescence is a complex and multistep biological process which cells can undergo in response to different stresses. Referring to a highly stable cell cycle arrest, cellular senescence can influence a multitude of biological processes—both physiologically and pathologically. While phenotypically diverse, characteristics of senescence include the expression of the senescence-associated secretory phenotype, cell cycle arrest factors, senescence-associated β-galactosidase, morphogenesis, and chromatin remodelling. Persistent senescence is associated with pathologies such as aging, while transient senescence is associated with beneficial programmes, such as limb patterning. With these implications, senescence-based translational studies, namely senotherapy and pro-senescence therapy, are well underway to find the cure to complicated diseases such as cancer and atherosclerosis. Being a subject of major interest only in the recent decades, much remains to be studied, such as regarding the identification of unique biomarkers of senescent cells. This review attempts to provide a comprehensive understanding of the diverse literature on senescence, and discuss the knowledge we have on senescence thus far.

## 1. Introduction

Since it was first identified and coined in the renowned Hayflick experiment in 1961, the concept of cellular senescence has raised considerable interest in the scientific community, not just for its experimental significance, but also, more meaningfully, its role in physiological processes [1]. The discovery of what is now known as the “Hayflick’s limit” had radically reformed the scientific dogma within Hayflick’s time, and stated that cultured cells generally could not divide indefinitely. Since then, the examination into senescence for the past decades has set the foundation for the understanding behind the cellular and molecular characteristics of senescence and has paved the way for senescence-based translational studies, albeit this research merely scratches the surface.

Senescence, which was originally characterised as a static cell fate, is now understood more as a complex and multistep biological process that cells can undergo—even in tumour cells which are characteristically immortal [2]. In simple terms, cellular senescence refers to a highly stable cell cycle arrest that acts as a defence mechanism in response to different stresses—these include oncogenic activation, tumour suppressor gene inactivation, oxidative stress, telomere attrition, mitochondrial dysfunction, as well as DNA-damage causing agents, such as irradiation and chemotherapeutic drugs [3,4]. In addition to a halt in cellular growth and division, phenotypical changes, including metabolic reprogramming (most prominently, the production and secretion of a complex array of factors collectively known as senescence-associated secretory phenotype (SASP)), chromatin rearrangement, and autophagy regulation, have been observed in senescent cells [5].

While senescence is often associated with aging and age-related diseases, senescent cells have an influence on a multitude of processes, such as embryogenesis and tissue remodelling, wound response, anti-cancer response, and tumourigenesis [2,6,7,8,9]. The role of senescence is thus dualistic; that is, senescence has both agonistic and antagonistic functions, and these functions are associated with the transient and prolonged presence of senescent cells, respectively. Indeed, it seems counterintuitive to the well-established dogma of evolution for a seemingly detrimental process, such as senescence, to be selected for and passed down from generation to generation. In 1957, however, the American evolutionist George C. Williams proposed the hypothesis of “antagonistic pleiotropy” in attempt to explain the evolution of aging, and this hypothesis has remained widely popular and as the primary explanation for senescence evolution since [10]. The theory posits that genes, which influence more than one phenotypic outcome (pleiotropy) and have beneficial effects early in life, only to be detrimental at a later age, can prevail in natural selection and thus be propagated. This has since been affirmed by progresses in senescence studies, which have uncovered the role of senescence in beneficial biological processes, such as limb patterning during foetal development and the prevention of tumour cell proliferation in early tumourigenesis.

### 1.1. DNA Damage Response

To further understand the presence of senescent cells in different physiological and pathological processes, studies on senescence have probed into the mechanisms by which cells gain senescence. It was found that senescence is often triggered by both intrinsic stressors (telomere attrition, oncogenic activation, oxidative damage, etc.) and extrinsic stressors (exposure to ultraviolet radiation and chemotherapeutic agents, etc.) that cause a persistent DNA damage response (DDR), which is an inherent cell DNA repair programme that is characterised ultimately by the activation of the p53-p21^CIP1^ pathway [11]. DDR activation can be either telomere-dependent or telomere-independent. Telomeres are several kilobase-long regions of DNA that cap the ends of chromosomes, and these have an imperative function in maintaining the integrity of DNA and the stabilisation of DNA molecules [12]. After every round of DNA replication, DNA molecules lose a short stretch of their telomeres (ranging from 50 to 200 base pairs) at the 3′ end, due to an end-replication problem in which the DNA polymerases are unable to faithfully replicate DNA molecules [13]. This progressive shortening of the telomeres has implications on the number of cycles that a normal cell can divide. As they reach their Hayflick’s limit, the integrity of DNA molecules is lost, as the chromosomal ends are exposed [14]. This intrinsic insult on the DNA molecules is detected by the DDR machinery and this activates replicative senescence [5]. On the other hand, telomere-independent DDR activation results from genotoxic stress that causes DNA breaks, and hence, “premature” senescence. For instance, in oncogenic-induced senescence (OIS), oncogene activation (the hyperexpression of gene products that promote cell proliferation) results in stalled replication forks with the increased usage of DNA replication origins, leading to DDR [15].

### 1.2. Senescence vs. Quiescence: Different Cell Cycle Arrests

While cell cycle arrest is a fairly common strategy in cells, and plays a crucial role in biological processes such as DNA replication, senescence is fundamentally unique from other cell cycle arrest mechanisms, such as quiescence. Although the G_0_ phase in the cell cycle (the resting state, when cells exit the cell-cycle and stop dividing) may be characterised by either type of cell arrest, senescence is essentially different, in that it is a permanent state of cell cycle arrest (although transient expression is possible); senescent cells are non-responsive to mitogenic factors and are unable to proliferate even in favourable conditions. Senescent cells are, at least naturally, also found to be resistant to apoptotic signals (which leads to apoptosis, an alternative cell fate that results in programmed cell death), a property which is conferred by the secretory factors of senescent cells [16]. However, quiescence is only a temporary state of arrest, and quiescent cells can re-enter and resume the cell cycle, given the right signals [17]. Logically, quiescence plays different biological functions from senescence. Most notably, quiescence has an imperative role in the maintenance of stem cell populations, by preventing the loss of genomic integrity that comes with the stress of replication, which is important for tissue homeostasis within organisms [18,19].

Despite much effort and traction in studies, senescence remains an inadequately understood concept, given that it has only garnered considerable attention in the last few decades. The difficulty in studying this cell fate is compounded by its paradoxical roles in both physiological and pathological processes, and the fact that its phenotypic expression is dependent on many factors, such as cell type, and the stressors that induce it. Much remains to be understood about senescence, including the detection of senescent cells. The search for a biomarker that is uniquely characteristic of senescence continues, as most phenotypic expressions of senescent cells are not exclusive and have limitations of some sort—even with the hallmark senescence-associated β-galactosidase (SA β-gal) activity, which is characterised by a significantly high level of β-gal activity that is detected at a suboptimal pH (pH 6.0) for the enzyme [20].

As headway is being continuously made in the study of cellular senescence, this review attempts to provide a comprehensive summary of the diverse literature and to gather the most important and fundamental knowledge we have on senescence thus far.

## 2. Senescence Biomarkers

**Figure 1:**
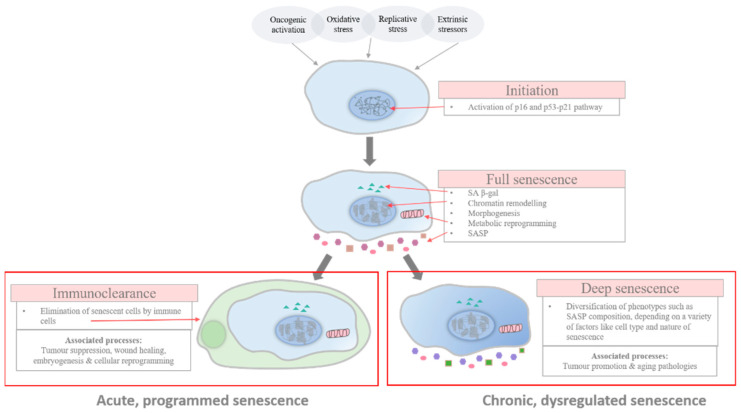
Stages of senescence. This diagram outlines the progression of senescent cells, from initiation to full senescence, and then resulting in one of two fates: clearance by the immune system or progression into deeper, chronic senescence. In most cases, the initiation of senescence is characterised by the activation of the p16 and/or p53-p21 pathway. The development into full senescence is then enacted by the extensive chromatin remodelling, occuring within the nucleus, which effectuates senescence metabolism, such as the production of SASP, and the irreversibility of senescence programme is enforced by the upregulation of p16. At the late stage, senescent cells are either cleared by the immune system in programmed senescence or persist into a state of deep senescence, in which their phenotype diversifies depending on multiple factors, such as cell type. Acute programmed senescence is associated with beneficial physiological process, such as tumour suppression and wound healing, while chronic, dysregulated senescence is associated with pathologies such as tumour promotion and aging.

More than just merely a descriptive state of being, cellular senescence is an intertwining of associated processes, a programme of dynamism, which is very similar to the course of cellular division. Even though it is poorly defined, senescence can generally fall into 3 stages: early, full, and late senescence (Figure 1). The initial stage of senescence is usually characterised by the induction of cell-cycle exit which is mediated by the p16 and/or p53-p21 pathway, in response to cell-cycle arrest signals such as DNA damage, although it has been demonstrated in some cases that senescence can proceed independently of p16 and/or p53-p21, such as senescence that is induced through the downregulation of histone acyltransferase p300 or BRAF^V600E^ oncogene activation [2,21,22]. The development into full senescence then involves progressive chromatin remodelling, which is mainly as a result of the downregulation of lamin B1, a major scaffolding protein of the nuclear envelope that plays numerous roles, including the regulation of chromosomal structures, which results in a vast change in the transcriptional profile [23]. Additionally, the upregulation of p16 has also been reported to be essential in enforcing the irreversibility of the senescence programme [24]. The downregulation of lamin B1 has been observed in multiple models of senescence, including replicative senescence, mitochondrial dysfunction-associated senescence, and Ras-induced senescence. Reportedly, this decline in lamin B1 is due to LC3-mediated autophagic degradation and mRNA regulation. In Dou et al., it was found that LC3, a nuclear autophagy protein, is crucial in inducing oncogenic-induced senescence through the mediation of lamin B1 degradation, and that the inhibition of autophagy or the LC3–lamin B1 interaction attenuates senescence [25]. Furthermore, Dreeson et al. had found that lamin B1 downregulation in senescent fibroblasts may possibly be mediated by miRNA (miR-23a) blocking, which prevents the translation of lamin B1 mRNA [26]. Additionally in Freund et al., lamin B1 mRNA levels were found to have decreased in an irradiation-induced senescent human fibroblast model, and that this decrease in lamin B1 was found to be dependent on p53 and Rb [27]. Rb is a tumour suppressor protein that plays a vital role in regulating the cell cycle. The association of hypo-phosphorylated Rb with activator E2F transcriptional factors, which regulate the promoter regions of genes that are involved in cell proliferation, including the lamin B1 gene, inactivates the E2Fs and prevents the expression of genes that are essential for the progression of the cell cycle. This profound chromatin remodelling, in turn, leads to the effectuation of various senescence programmes, those most notably including SASP production, increased SA β-gal activity, and mitochondrial metabolism. However, the role of lamin B1 in cellular senescence must be further studied, as there have been contradictory experimental results reported. Both lamin B1 overexpression and its reduction have been found to induce senescence in fibroblasts, but the overexpression of lamin B1 has also been associated with an increased proliferative capability and a delay in the onset of senescence [23,26,28].

In the full stage of senescence, senescent cells may be scheduled for immunoclearance or may persist further into a deeper state of senescence through the continuous alterations in their phenotypic expression via genetic and epigenetic changes, such as histone proteolysis [29,30,31]. In this late stage of senescence, phenotypic diversification is established and the differential expression of phenotypes, which include changes in the composition of SASP secretion, can be observed across different pathophysiological contexts [32,33].

While the categorisation into different stages reflects the progression and heterogeneity of senescence, there is no substantive proof of a link existing between the different phases and their impact on the pathophysiological functions of the senescent cells, however intuitive associations can be made. Despite so, connections have been made between the kinetics of senescence response and pathophysiological consequences [5]. Acute senescence, relating to a programmed strategy in cells in response to distinct stressors which is established transiently before being scheduled for self-orchestrated clearance, is believed to be implicated in homeostatic biological processes, such as embryonic development and wound healing. However, chronic senescence is associated with the prolonged exposure to cell damaging stressors, which causes cell cycling to be no longer viable and favourable, due to the tremendous burden placed upon reparative and regulatory pathways to maintain the cell’s integrity. Unlike acute senescence, chronic senescence is not a programmed response—it is persistent and is believed to play detrimental physiological roles, such as in aging and aging-related diseases.

Given the diverse phenotypic expression of senescent cells, there have been expectations of the discovery of truly unique markers that can help to identify cells undergoing senescence efficiently and definitively. However, efforts in trying to characterise senescent cells so far have come up short, for none of the biomarkers that have been discovered are exclusive to senescence, and hence cannot be used in isolation. While the search will continue, other studies have also attempted to approach this from different perspectives and have theorised on unique models that can be used to characterise senescence, based on the knowledge gathered to date. For example, an article in the recent literature proposed a novel interpretation which characterises the induction of senescence by the presence of seemingly conflicting signals, that is, factors for both cell expansion and for cell arrest [34]. Nevertheless, most of these proposed models remain hypothetical and require further investigation. 

Although the phenotypic traits that have been identified so far are not distinguishing features of senescence, they constitute important indicators of senescent cells, as these expressions and alterations in cellular metabolism drive the maintenance of the senescence programme and its pathophysiological functions.

### 2.1. Senescence-Associated Secretory Phenotype (SASP)

The most notable attribute of senescence metabolism is, most likely, the production and secretion of a combination of compounds that include cytokines, chemokines, extracellular matrix proteases, growth factors, and other signalling molecules. Referred to on the whole as the senescence-associated secretory phenotype, the SASP is one of the more, if not the most, important modulator of senescence, having implications for the progression and maintenance of senescence, the immune modulation of senescent tissues as well as the pathophysiological manifestations [5,35,36].

The SASP is thought to help maintain senescence growth arrest through an autocrine positive feedback loop. It has been shown that chemokine signalling, mediated by CXCR2-binding chemokines in the SASP, contributes to this self-amplifying growth arrest mechanism [37]. Interestingly, in addition to cell autonomous regulation, senescent cells can exhibit non-cell autonomous effects, mainly through the SASP, although this is also possible through other, less-studied ways, such as juxtacrine NOTCH/JAG1 signalling or cargo transfer via a cytoplasmic bridge. Interleukin-1 (IL-1), one of the most prominent proinflammatory cytokines that is found in SASP, has been linked to this paracrine activity of senescent cells, in which it can induce normal neighbouring cells to enter the senescence programme [38]. Other than signalling factors, senescent cells also express proteases (matrix metalloproteinases), growth factors (insulin growth factor-binding proteins), and non-protein macromolecular elements (nitric oxide and reactive oxygen species) that may mediate autocrine/paracrine activities through different pathways.

However, the SASP has also been found play an active role in mediating the clearance of these senescent cells by engaging the natural immune system through immunosurveillance–immunoclearance. Some components which are produced in the SASP of senescent cells attract and signal cells of the immune system, such as macrophages, which help to detect and eliminate the senescent cells in programmed senescence, such as the anti-tumourigenic response [30]. Depending on the context, different ligands are produced in the SASP, which will implicate different aspects of the immune system. For example, it has been documented in a mouse model that the senescence of activated hepatic stellate cells (HSCs, which contribute to fibrosis in response to tissue damage in the liver) help to ameliorate liver fibrosis by engaging natural killer cells (NK cells) in the clearance of these HSCs [29]. The senescent HSCs were found to upregulate the expression of the NKG2D receptor (cell surface receptor of NK cells) ligands, such as MICA and ULBP2, which recruit NK cells in the immunosurveillance–immunoclearance of HSCs [39]. In another instance of the oncogene-induced senescence of hepatocytes, the development of murine hepatocellular carcinoma was blocked via the activation of the CD4^+^ T-cell-mediated immune response, which is known to engage macrophages [30]. The immunomodulatory SASP factors have also been found to play an important role in the progression of tumours. For example, in hepatocellular carcinomas, it has been found that CCL2, released by oncogene-induced senescent hepatocytes, may result in the attraction of CCR2^+^ immature myeloid cells, which differentiate into macrophages to clear precancerous senescent hepatocytes, but may also promote tumour growth by inhibiting NK cells in the presence of established hepatocellular carcinoma cells [40].

The SASP can be regulated at multiple levels. Other than transcriptional control, SASP expression is regulated at post transcriptional (mRNA stability), translational and epigenetic levels as well [5]. Signalling pathways such as DDR, p38 MAP kinase (involved in cell differentiation, apoptosis and autophagy), and cGAS/STING (involved in the detection of DNA in the cytosol) have also shown to play a part in the transcriptional control of SASP through the activation of the transcription factor NF-κB, an important regulator of many cell responses to harmful stimuli which directly controls the expression of key proinflammatory interleukins, as well as amplifies senescence activity in an autocrine manner [37,41,42]. Another regulator of note is mTOR, a serine/threonine protein kinase, which helps to regulate the stability of SASP mRNAs [43]. The mTOR protein kinase activates the translation of MAP kinase-activated protein kinase 2 (MAPKAPK2) which then binds to and inhibits a SASP mRNA-degrading protein, ZFP36L1.

While the SASP is highly conserved within the same types of tissues, there exists varying features amongst SASP of different forms of senescence, even though they do show some degree of conservation, especially within the proinflammatory components [5]. For example, in fibroblasts, cells that undergo OIS appear to express factors such as IL-6 and CXCL1 more strongly than cells that undergo replicative senescence. This suggests that senescence stressors may have implications on the characteristics of individual cases, and this may be an avenue for future studies on the characterisation of senescence. However, the use of SASP components as unique biomarkers is still limited, because the SASP composition is circumstantial and the compounds are not unique to the senescence programme. The classical SASP factors and their examples are described in Table 1.

### 2.2. Cell Cycle Arrest Factors—p16 and p53-p21 Pathways

DDR is an inherent cellular mechanism that leads to the activation of a number of cell strategies (one of which being senescence) in response to DNA damage, through the activation of the p53-p21 axis [11]. Upon DNA damage, a signalling cascade involving ATM and ATR (serine/threonine protein kinases) is transduced in order to activate checkpoint kinases (CHKs), which in turn activate the p53-p21 pathway. p53 is an important tumour suppressor protein that induces the transcription of p21, a cyclin kinase inhibitor, which inhibits CDK2 and, as a result, prevents the progression from the G1 to the S phase of the cell cycle. p21 also promotes senescence through the downregulation of mitotic genes, such as CCNE2, and the upregulation of senescence-associated genes, such as fibronectin-1 [51]. In addition, p53 and p21 (and p16) may also inhibit apoptosis and thus favour senescence, which is discussed in Section 2.5 below.

While the p53-p21 pathway is an important mechanism through which senescence is induced, p53-deficient cells have also been observed to be capable of senescence through the p16 pathway. The p16 pathway has been discovered as a distinct, backup senescence mechanism in p53-proficient cells, which allows the reversibility of senescence when p16 is low, but not when it is present at high levels [24,52]. However, it has also been suggested that for the persistent maintenance of cell cycle arrest, p16 activation is required [53]. In the presence of long-lasting or extremely damaging stressors, the INK4/ARF gene is activated. The INK4/ARF gene encodes for three tumour suppressor proteins: ARF, p16, and p15INK4b. While ARF inhibits the p53-negative regulator MDM2 and prevents p53 degradation, p16 is responsible for the inactivation of CDK4/6. CDK4/6 plays a vital role in the activation of E2F cell-cycle transcription factors. When active, CDK4/6 mediates the partial phosphorylation of Rb, a tumour suppressor protein which is associated with the E2F transcription factors in their inactive state, which, in turn, results in the release and activation of the E2F transcription factors to allow cell cycle progression [54]. The inhibition of CDK4/6 by p16 thus prevents the activation of these transcription factors and prolongs cell cycle arrest.

However, the expression of these arrest factors may not occur in all types of senescence, and is not always dependent on DDR. For example, Storer et al. found that p16 and DNA damage markers are absent in developmental senescence. Only p21 was expressed in the developing embryos in this study, which was also independent of p53 [55].

While the p16 and p53-p21 cell arrest pathways are crucial in leading to senescence in most cases, the use of the compounds which are associated with DDR, such as gamma-H2AX (which remodels the chromatin around double stranded breaks), ATM/ATR, and CHKs, as markers of senescence is difficult. The activation of these pathways does not always indicate the transition into senescence, as other mechanisms can also become activated [56].

### 2.3. Senescence-Associated β-Galactosidase (SA β-gal)

One of the more distinguishing features of senescent cells, SA β-gal expression, is currently considered to be the hallmark of senescence, and thus the more indispensably used marker. SA β-gal, however, is not a unique isoform of the enzyme that is only found in senescent cells. Instead, this SA β-gal is the gene product of the GLB1 gene, which is associated with lysosomal β-galactosidase [57]. It has been found that this lysosomal, endogenous β-gal activity is significantly increased in senescent cells when compared to normal cells as a result of an increased lysosomal content in senescent cells [57,58,59,60,61]. The β-gal activity detected in senescent cells using histochemical assays such as flow cytometry at pH 6.0, which would otherwise not be detected in non-senescent cells as the optimal pH for the enzyme lies around pH 4.0–4.5, is thought to be due to the sheer hyperproduction of the enzyme.

Despite this, there are some limitations to using SA β-gal as the sole marker. SA β-gal, although usually present in huge amounts, appears to be non-essential in senescence. Cells which are deficient in the gene encoding this enzyme have also been found to undergo senescence unhindered [57,61]. Also, there is evidence that SA β-gal can be detected in some cells which are not undergoing senescence, such as in contact-inhibited or serum-starved cells grown in tissue cultures [62]. For example, in ageing mice, it has been found that the majority of the SA β-gal-positive cells that accumulate are not senescent cells, but instead are macrophages with senescence-like characteristics, as a result of physiological reprogramming [63]. Thus, in order to ascertain senescence in cells, other markers are still required to complement the SA β-gal assay.

### 2.4. Morphogenesis and Chromatin Remodelling

In addition to a change in their chemical expression, senescent cells undergo morphogenesis as well. Generally, senescent cells are enlarged, flattened, vacuolised and, at times, multinucleated. These morphological changes have been linked to caveolin-1, which regulates focal adhesion kinases and the actin cytoskeleton in senescent cells, although the functional significance of these changes is still largely unknown [64].

Chromatin remodelling, which results in the formation of senescence-associated heterochromatic foci (SAHFs) and can be visualised under DAPI nucleic acid staining, has also been observed in OIS cells. This structure has been found to be enriched with proteins associated with gene repression, such as heterochromatin protein 1 (HP1), through the spatial repositioning of the proteins from pre-existing heterochromatins. Although its exact function is still unclear, it is believed to enact the irreversibility of senescence phenotypes by burying pro-proliferative genes and preventing access to them [65]. As with the senescence-associated chemical phenotypic traits, this structural feature of senescence, alone, is not a good indicator of senescence, as it is not consistently observed across all senescent cells [66].

### 2.5. Apoptosis Sensitivity

Apoptosis refers to a type of programmed cell death that plays an integral role in numerous biological processes and in maintaining homeostatic cellularity [67]. For example, during human embryo development, the separation of the fingers and toes relies on the apoptosis of cells between the digits; the absence of or a defect in the apoptosis programme results in syndactyly, a condition in which the adjacent digits are fused together. In general, caspase-dependent apoptosis (although caspase-independent apoptosis is possible too) can be effectuated in two major ways: the extrinsic pathway, which is mediated by cell-surface death receptors, and the intrinsic pathway, which involves the mitochondria. Regardless of which, both types of pathways result in the activation of enzymes known as caspases, which ultimately cause the degradation of cellular components. Once thought to be an irreversible process once it was committed to, recent studies have discovered that the reversion of dying cells to a pre-apoptotic state, termed “anastasis” is a crux in cancer relapse, amongst other outcomes. This ability has been linked to the heterogeneity of mitochondria across and within cells (such as variations in cardiolipin content, which facilitates the integration of apoptosis signals), which results in different degrees of “activation” [68,69]. This prevents the induction of complete apoptosis, and the surviving cancer cell can undergo anastasis by the upregulation of genes responsible for a variety of processes, such as in proliferation [70]. However, these cells exhibit genomic alteration, enhanced tumorigenicity and metastatic capacities. Genomic alteration is likely caused by (sublethal) caspase-activated endonuclease activity and the degradation of histones, which de-compact chromatin for DNA repair, as well as promote unwanted mutations [71]. Ethanol treatment has been observed to result in the formation of micronuclei, which is evidence of chromosomal aberration in some anastatic cells, and these cells exhibit anchorage-independent growth. Moreover, migration-promoting genes such as MMPs have also been found to be upregulated in late anastasis, which can promote cancer cell metastasis [70].

As an alternate cell fate, apoptosis can be a choice of tissue salvage in which cells engage in response to cellular stress, instead of senescence. So how did senescence as a mechanism for salvation come to be retained, when apoptosis seemingly appears to be far superior amongst the two from an evolutionary standpoint, given that apoptosis can lead to the direct removal of rogue cells, and that senescence has a hand in numerous pathologies? What decides whether cells undergo apoptosis or senescence? While it is clear that apoptosis and senescence share a certain degree of commonality, no definitive answers have been established to these questions, which confounds the examination into senescence. In the 2014 review by Childs et al. which explored the intricacies of these two cell fates, it was suggested that senescence could possibly be advantageous over apoptosis due to its ability to exert both cell-autonomous and non-cell autonomous effects [72]. For example, unlike apoptosis which “can be viewed almost solely as a cell-intrinsic mechanism”, senescence may more effectively clear dysfunctional cells, such as in early tumours, by working in conjunction with the immune system. Rather than relying on each cell to enter apoptosis, which may be hampered by impairments due to damage, senescence can exert a more profound effect that extends to neighbouring cells, either by directly causing their immunoclearance, or by signalling them to enter into senescence through the SASP, requiring only minimal cells to attain senescence.

p53 and its downstream targets have a hand in regulating the cell fate. On one hand, p53 mediates apoptosis by upregulating pro-apoptotic factors, such as PUMA and NOXA; on the other, p53 may also upregulate anti-apoptotic proteins, including p21 and DNAJB9 [73,74]. p21, in addition to promoting SIPS, prevents apoptosis in a few ways. Firstly, it may downregulate p53 by inhibiting the p14–MDM2 interaction, which allows MDM2, the negative regulator of p53, to degrade p53. Secondly, p21 also may inhibit apoptosis-related proteins that are involved in apoptosis, such as caspases and ASK-1 [75]. Lastly, p21 may modulate the transcription of anti-apoptotic (eg., prosaposin) and pro-apoptotic genes, as well as inhibit apoptosis-inducing transcription factors such as MYC.

In addition, p16 also has the ability to modulate apoptosis. Under certain circumstances, p16 may repress apoptosis, such as in UV-induced intrinsic-mitochondrial apoptosis, by downregulating the pro-apoptotic Bax protein, but is also known to induce cisplatin-mediated apoptosis by downregulating BCL-2 [76]. Furthermore, p16 also exists in a complex mutual regulatory loop with p53. It has been reported that while p16 may downregulate p53, by repressing its transcription and promoting its degradation via MDM2, p53 has been suggested to also downregulate p16, through the upregulation of the p16-repressor, Id1 [77].

Not surprisingly, the cell type is likely one of the factors that determines the stress response. It has been observed that cells preferentially engage in one mechanism over the other [78]. In addition, the severity and type of stress that is experienced also determines the fate of cells. It has been observed that moderate doses (1–8 Gy) of ionising radiation tend to cause stress-induced premature senescence (SIPS) in p53-proficient human fibroblast strains and numerous solid tumour-derived cell lines, rather than apoptosis. In the case of UVC, it was observed that in human fibroblasts, moderate doses (<15 J/m^2^) resulted in a small increase in p53 (three-fold), the upregulation of p21 and the engagement of SIPS, while high doses such as 30 J/m^2^ resulted in a large increase in p53 (20-fold), an inhibited p21 expression, and lead to the engagement of apoptosis [79,80]. Similarly, chemotherapeutic agents such as cisplatin are also known to induce apoptosis only at high doses, and induce SIPS mainly at moderate doses. Generally, apoptosis is induced when the stress experienced is graver (for example, in the presence of higher doses of DNA-damaging agents), while senescence is induced when it is less severe (lower doses of DNA-damaging agents) [81]. However, it has also been shown that certain genotoxic agents, such as busulfan, induce senescence regardless of dosage, and this implies that the nature of the stress also comes into play [82].

Despite the immense stress that is experienced by senescent cells, which should theoretically signal their death, some senescent cells (such as in fibroblasts and keratinocytes) are instead more resistant to apoptosis. This resistance of senescent cells to apoptosis is enforced by various pathways which are collectively known as the senescent cell anti-apoptotic pathways (SCAPs) [16]. One of these pathways involves the BCL-2 family of regulator proteins, which control apoptosis at the mitochondria, and are central to this characteristic of senescent cells. Evidence reported in various studies has demonstrated that BCL-W, BCL-XL and BCL-2, anti-apoptotic factors of the BCL family, were upregulated in senescence-induced cells, and that the knockout of these genes led to a decrease in the successful induction of senescence in cells [83,84,85]. However, it is also important to note that there are exceptions. For example, senescent endothelial cells show an increased sensitivity to apoptosis instead, and express lower levels of BCL-2 [72]. This variation is attributed to the differences in the mechanisms that are present in different senescent cells, and is associated with how they modulate pro-survival and pro-apoptotic factors.

### 2.6. Metabolic Reprogramming

When cells undergo senescence, their metabolisms are also reprogrammed in order to aid in the maintenance of the senescent state. Most notably, senescent cells have been found to have increased glycolysis, mitochondrial metabolism, and autophagy activities [86]. It is believed that this elevation in metabolic activities is an adaptation to provide senescent cells with sufficient amounts of nutrients and energy to facilitate the expression of highly active senescent phenotypes, such as the production of the SASP. Besides increasing the amount of ATP through glycolysis and mitochondrial metabolism, autophagy in senescent cells has been found to be coupled to protein synthesis so as to overcome the shortage of amino acids [87]. In addition, autophagy is also thought to help in maintaining the survival of senescent cells. Proteotoxic ER stress, caused by the build-up of unfolded proteins (as a result of hyperactive SASP production), and oxidative stress, caused by ROS-damaged biomolecules, is believed to trigger the unfolded protein response (UPR) in senescent cells, which in turn activates the autophagy machinery [86,88,89,90]. Autophagy clears and alleviates the cells of these stresses that can otherwise promote apoptosis, thus maintaining cell survival.

**Figure 2:**
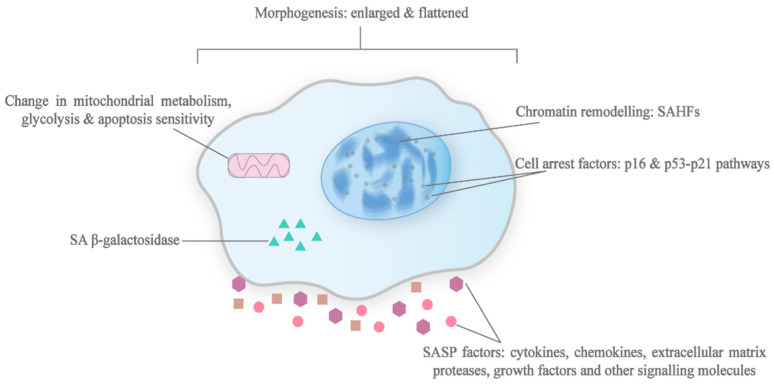
Characteristics of senescent cells. This diagram depicts the typical phenotypic changes that are associated with senescence. In most cases, senescent cells exhibit an enlarged and flattened morphology, chromatin remodelling which leads to the formation of SAHFs, production of the SASP and cell arrest factors like p53, increased lysosomal mass containing β-galactosidase, and metabolic reprogramming.

However, the role of the mitochondrial metabolism in senescent cells has been disputed. Senescent cells that are depleted of mitochondria have been shown to exhibit an elevated glycolysis activity and higher ATP levels on the contrary to other evidence [86]. Despite this, senescence becomes inviable in these cells. It has been proposed that the mitochondrial metabolism has a considerably more important role in enforcing the senescent state via a positive, DDR-dependent feedback loop, which involves excessive ROS production as a result of mitochondrial dysfunction, caused by an impairment to mitostasis and the mitochondrial dynamics (such as decreased mitophagy), that is characteristic in senescent cells [5,91]. The characteristics of senescent cells are illustrated in Figure 2.

## 3. Physiological and Pathological Roles of Senescence

### 3.1. Embryogenesis

The importance of programmed cellular senescence can be observed as early as mammalian embryonic development [55]. Senescence has been observed throughout developing embryos, such as in the mesonephros and endolymphatic sac, and has been found to play a significant role in limb patterning and tissue growth [7,55,92]. While it is only transiently present during a certain window (detected usually through SA β-gal staining at pH 5.5) before being cleared by macrophages, developmental senescence is a tightly regulated and programmed process, which is characteristic of beneficial senescence programmes. The senescent embryonic cells, however, show an interesting difference in that they are highly dependent on p21, but not p53 or p16. p21 in developmental senescence is upregulated in a p53-independent manner, relying on the TGF-β/SMAD and PI3K/ FOXO signalling pathways instead [7].

Despite the role of senescence in the embryonic development process, the gravity of the consequences for impairing p21 in embryos appears to be rather trivial. p21-deficient mouse models are considered to be developmentally normal, despite a reduction in SA β-gal-positive cells during embryogenesis, and this has been attributed to the resilience of embryos and their ability to engage alternate mechanisms for remedy [55].

### 3.2. Wound Healing & Fibrotic Response

Even in wound healing, senescence can play a wide variety of roles to promote tissue repair through the SASP. Besides stopping the proliferation of damaged cells and supplying the SASP growth factors that stimulate repair, senescence has crucial roles in wound closure and tissue remodelling to prevent fibrosis [93,94]. The SASP secreted by senescent cells at wounds contains the platelet-derived growth factor AA (PDGF-AA), which induces the surrounding fibroblasts to differentiate into myofibroblasts [8]. These myofibroblasts are responsible for hastening the repair by contracting the wounds. Furthermore, SASP factors, such as MMPs, also help to prevent fibrosis caused by the deposition of extracellular matrix components, which, although essential for tissue repair, can impede restoration in excessive amounts by resolving fibrotic scars [93]. These senescence-associated, fibrosis-limiting responses have also been reported in the liver and within infarcted hearts to prevent the development of function-impairing fibrosis [29].

More importantly, however, is the self-clearance mechanism of senescent cells, which is affected by the activation of the immune system, for without their proper removal, this then leads to their accumulation and ultimately impedes any healing [93]. Pro-inflammatory factors, such as chemokines in the SASP, are responsible for recruiting macrophages so as to clear senescent cells. An attest to the cruciality of the tight regulation and removal of senescent cells for any beneficial gain is shown in idiopathic pulmonary fibrosis (IPF), a chronic lung disease, in which senescence plays a contradictory role in the fibrosis response [95]. It has been found that in bleomycin-induced IPF mouse models, senescent cells accumulate and contribute to the condition by expressing profibrotic SASP instead of promoting healing, while their elimination improves the condition.

### 3.3. Cellular Plasticity & Reprogramming

The expression of the Yamanaka reprogramming factors (OCT3/4, SOX2, c-MYC, and KLF4) in cells can result in reprogramming-induced senescence, which is activated through p16 and p21 [96]. While the cells that undergo reprogramming-induced senescence do not undergo reprogramming themselves (due to the repression of senescence mediators), they do encourage the reprogramming of their neighbouring cells through their SASP. In fact, senescence is an obstacle to reprogramming in cells, as evidenced by experiments that have shown an increase in the reprogramming efficiency of cells when p53, p21, and p16 genes are silenced [97]. However, it is believed that senescence is still activated (in non-reprogramming cells) under reprogramming signals, as IL-6, which is produced by these senescent cells, helps in enhancing the c-MYC activity and in the alteration of the stemness of neighbouring cells [93,98]. Similar to the other beneficial roles of senescence, it is essential that the senescence kinetics are transient for the regeneration programme to be implemented, otherwise the prolonged exposure of non-senescent cells to the SASP may promote senescence in these cells instead of reprogramming.

### 3.4. Ageing & Ageing Related Diseases

Ageing, defined as the gradual deterioration of tissue/organ functions that are essential for survival and fertility, is a process caused by a myriad of factors, including, but not limited to, the exposure to oxidative stress and telomere attrition, and is associated with senescence, apoptosis, and other cellular responses that cause dysfunctionality in multicellular organisms [94,99]. Regarding senescence, multiple studies have established a strong link between the accumulation of senescent cells with ageing-associated degeneration in experimental models. For example, in Maier et al., mouse models with a hyperactive p53 activity displayed the rapid accumulation of senescent cells and signs of (premature) ageing, such as loss of fertility, retarded wound healing, and reduced hair growth, to name a few [100,101].

Similar to other physiological and pathological processes, there are multiple roles of senescence in ageing pathology. Firstly, senescence can drive ageing by depleting stem cells, which are the source of cell replenishment, as they accumulate DNA damage [94,102]. For example, in Sousa-Victor et al., satellite cells (stem cells responsible for the regeneration of skeletal muscle fibres) in aged mice were found to enter senescence and lose their regenerative function [103]. With the reduction in stem cells, senescence thus impedes tissue repair in aged organisms and interferes with the restoration of tissue function in these organisms.

Secondly, the decline in and the dysregulation of the function of the immune system due to senescence in immune cells, termed “immunosenescence”, is believed to result in the less effective clearance of senescent cells in the body [104]. The diminishing of immune cells, such as the macrophages which are involved in the surveillance and clearance of senescent cells, allows senescent cells to accumulate and partake in ageing pathologies. As established, the persistence of senescence causes a problematic decline in tissue function by interrupting tissue homeostasis, interfering with important biological processes such as cell differentiation, and even promoting tumourigenesis [2,78].

Moreover, immunosenescence, together with the SASP of senescent cells, also contribute to a persistent, low-grade inflammation known as “inflamm-ageing” in organisms, which promotes ageing and results in tissue damage [102,105]. Senescent cells secrete a wide range of factors, including pro-inflammatory chemokines and cytokines, which attract and induce the immune system [38]. However, when these inflammatory signals cannot be resolved by the declined immune system as a result of the senescence of immune cells, inflammation is amplified, which results in a persistent chronic cycle and in the accumulation of senescent cells [106]. Chronic inflammation can hinder with physiological processes and contribute to pathologies such as atherosclerosis, type II diabetes, and osteoporosis [105].

### 3.5. Cancer

Unsurprisingly, senescence has a hand in both tumour suppression and tumour promotion when it comes to cancer development, a complex process which is influenced by both internal and external factors [107,108,109,110,111,112,113,114,115]. While senescent non-tumour cells have the potential to promote cancer by influencing the tissue microenvironment, senescence in tumour cells is one of the strategies that impedes the unregulated proliferation of these dysregulated cells.

In particular, OIS, which is brought about by the activation of oncogenes and is regulated mainly by the Rb and p53 proteins, has been identified as the anti-tumour mechanism that is employed to inhibit the growth of tumour cells that are dysregulated in early tumourigenesis [116]. Studies have shown that in neoplastic tissues in which oncogenic activation and senescence markers have been identified, the aberrant cells rarely become malignant and lead to cancer formation [117]. In support of this, tissues in which the senescence response has been inhibited have shown a propensity for malignant tumour development [118,119].

**Figure 3:**
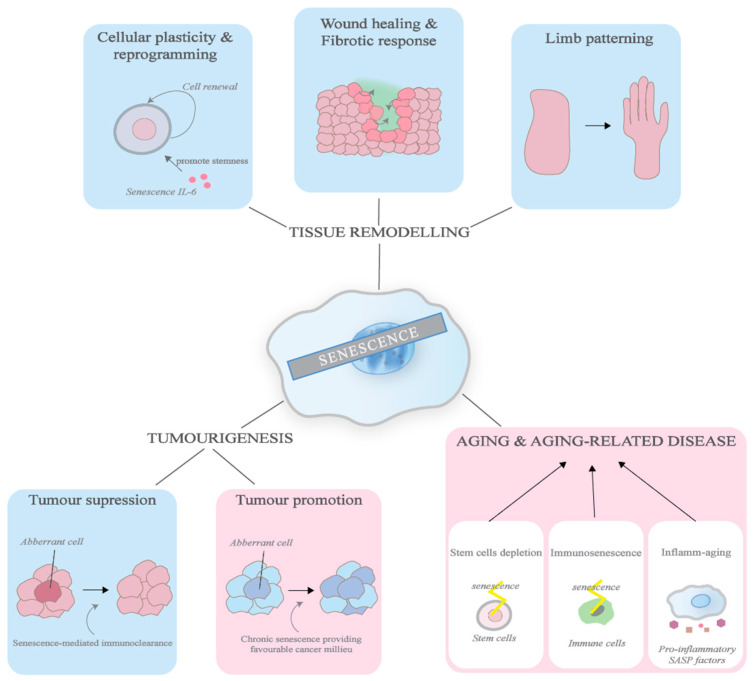
Consequences of senescence. This diagram illustrates the physiological and pathological roles senescence plays. The effects in the blue boxes indicate the beneficial processes while the ones in red indicate the detrimental processes. In physiological processes, senescence helps in tumour suppression, the promotion of cellular regeneration, wound healing, and limb patterning. In pathological processes, senescence exacerbates tumour development and partakes in ageing pathologies by depleting stem cells and immune cells, as well as promoting inflammation.

Nevertheless, persistent senescence promotes cancer formation. Senescence is believed to encourage malignant tumour growth mainly through the paracrine signalling of the SASP components [116]. The ECM has an integral role in influencing tumour behaviour, and has been found to evolve in the course of the disease to adapt to the progression of the tumour [120]. Senescent cells can alter both the ECM and tissue microenvironment and provide a favourable milieu for tumour progression. For example, IL-6, a significant determinant of cancer progression that is produced by senescent mesenchymal stem cells, has been found to promote the growth and metastasis of breast cancer cells [121]. Figure 3 illustrates the consequences of senescence.

Of a notable significance, cancer cells have been found to be able to undergo senescence as a result of therapeutic treatments, such as chemotherapy and radiation, in a process known as “therapy-induced senescence” (TIS), although the term is not limited to cancer cells in particular and can occur in non-tumour cells [96,116]. Treatments such as doxorubicin and ionising radiation can lead to senescence in a variety of cancer cells, including colon carcinoma, breast cancer, and fibrosarcoma, either directly or through a paracrine influence. Other experiments have shown that etoposide treatment resulted in senescence in a variety of cell lines, including HEK293 T human embryonic kidney cells, U2OS osteosarcoma cells, and H1299 NSCLCs [122]. This has implications for cancer patients, as the persistence of TIS cells has been linked with the aggravation of the side effects of treatments, as well as the relapse of cancer. Clinically relevant doses of chemotherapeutic agents have been found to ineffective in inducing complete cell death, but instead give rise to a few outcomes that allow the survival of cancer cells and contribute to relapse, including polyploidisation, anastasis and senescence.

The persistence and accumulation of senescent cells following cancer therapy may potentially contribute to health problems, including relapse, that arise post-treatment. It has even been reported that doxorubicin-induced senescent breast cancer cells can develop the ability to engulf neighbouring cells, so as to appropriate nutrients for their own survival [123,124]. It is thought that the SASP, as well as the transcriptional changes in the senescent cells, give rise to these detrimental effects. The SASP of these senescent cells is believed to promote the proliferation of non-senescent neighbouring (tumour) cells, as demonstrated in melanoma and prostate cancers by Sun et al. and Ewald et al., and has been shown to induce an epithelial-to-mesenchymal transition (EMT), which potentially confers tumour cell mobility and, consequently, causes metastasis [125,126]. Additionally, the SASP has also been shown to impede the immunosurveillance and immunoclearance of tumour cells, contrary to the immune system-stimulatory characteristics mentioned earlier: in one study, senescent stromal cells were found to recruit myeloid-derived suppressor cells that attenuated the CD8^+^ T cell response and promoted tumour growth [45]. Similarly, the drastic change in the transcriptional profile of senescent cells has also been shown to limit the success of current anti-cancer therapy—the upregulation of pro-survival, anti-apoptotic signals such as BCL-2, as a result of transcriptional changes, is thought to desensitise senescent cells to apoptosis, which diminishes the effectiveness of anti-cancer therapies that employ apoptosis-inducing strategies [127,128].

Interestingly, it has been shown that TIS tumour cells are able to escape the senescence programme and re-enter the cell cycle. Multiple in vitro studies have demonstrated that senescent tumour cells that are induced by chemotherapeutic drugs or ionising radiation regain the ability to proliferate and form colonies in culture [129,130,131,132]. For example, in Was et al., a six-fold increase in cell number was observed in a population of doxorubicin-induced senescent human colorectal carcinoma HCT116 cells two weeks after the removal of the drug [133]. Although not much is known about the mechanism behind this phenomenon, it has been reported that, in the instance of camptothecin-induced senescent non-small cell lung carcinoma cells (NSCLCs), Cdc2 and its downstream effector, survivin, were found to play a critical role in this escape ability in vitro. This ability of TIS tumour cells to exit the senescence programme is thought to cause the relapse of cancer in patients [129,134]. It has been shown that these cells acquire a phenotype unlike their pre-senescent state, with an increased metastatic potential and stem cell-like characteristics (e.g., the expression of the pluripotency-associated transcription factor NANOG), which results in aggressive proliferation. In Saleh et al., it was observed that senescent H460 cells which were implanted in mice resulted in tumour formation in the mouse models, suggesting that the senescent tumour cells that escaped the senescence programme can cause tumour development in vivo [131]. In a study which investigated breast tumour samples, 15 out of 36 samples from patients who underwent neoadjuvant chemotherapy showed an increase in SA β-gal when compared to 2 out of 20 samples from patients who did not undergo treatment [135].

More recently, the ability of TIS cells to escape senescence has been linked to polyploidisation, the event in which cells gain more than 2 sets of chromosomes. It has been found that whole genome duplication is common in cancer (37% of solid tumours) and that anticancer therapies significantly increase the frequency of polyploid giant cancer cells (PGCCs) [136,137,138]. This phenomenon allows for the survival of cancer cells, as PGCCs can undergo depolyploidisation to produce progeny, and this is associated with a poor prognosis in patients [139,140]. Perhaps polyploidisations may explain why senescent tumour cells are able to regain their proliferative capability and why these daughter cells exhibit a different phenotype, through the asymmetric, non-mitotic cell division that is similarly observed in PGCCs. PGCCs have been shown to produce near-diploid progeny from a process termed the giant cell cycle, in which the giant nucleus of a PGCC gives rise to smaller nuclei through nuclear budding, nuclear fragmentation, or nuclear fission [141]. This is then followed by cyto-fission that gives rise to diploid daughter cells, which gain a mitotic capability and have stable genomes. A few pieces of evidence have established the link between TIS escape and polyploidisation, including Puig et al., in which a rat model that was injected with a colon cancer cell line and treated with cisplatin produced giant cells which were positive for SA β-gal [130,142]. However, the exact mechanism of division has not been studied. While anticancer treatments may induce either senescence or polyploidy, or both processes, and the sequence in which they are acquired may differ, it has also been suggested that cancer relapse is dependent on polyploidy rather than senescence, thus reinforcing the idea that TIS may very likely use the polyploidy strategy to escape [143,144].

## 4. Therapeutic Significance

With the discovery of the roles that senescence plays physiologically, studies emerging in recent years have placed much focus on exploring and developing treatments that target senescent cells. Implicated in numerous and diverse physiological/pathological processes, the promise of treatments targeting senescence is apparent. Pro-senescence treatment for cancer therapy and senotherapy for combating ageing and its associated diseases are both emerging fields of studies that have the potential to revolutionise the current (flawed) treatment strategies and possibly allow us to attain longevity.

### 4.1. Senotherapy

Senotherapy is concerned with the development of therapeutic strategies to slow the ageing process and alleviate its associated diseases by preventing, eliminating, or reversing senescence in cells [145]. Although much of this study is still exploratory, and no drugs have been approved for clinical use, many different strategies have been identified. Central to all is the use of senolytics. Senolytic therapy is one of the more, if not the most, rapidly developing strategy for senotherapy. Senolytic agents are a class of small molecules that have been found to be able to selectively induce the apoptosis of senescent cells by interfering with the SCAPs. Most senolytic drugs that have been identified so far are repurposed anti-cancer drugs, such as the likes of dasatinib and quercetin (used in combination)—the first few senolytics to be discovered. The combination of these two drugs has been widely demonstrated to induce the apoptosis of senescent cells in cultured human tissues and in mouse models [146,147,148]. In one of the mouse models, the bi-weekly administration of dasatinib and quercetin on aged mice showed a 36% higher median lifespan, while the mortality hazard was reduced to 64.9% [145]. This potential of the senolytic drugs has also been replicated in clinical trials—in the first-of-its-kind Phase 1 clinical trial, the administration of dasatinib and quercetin on patients with idiopathic pulmonary fibrosis (IPF; a lung disease in which scarring of the lungs reduces their function) showed a significant improvement in physical functions (such as walking endurance) in the participants [149]. The following subsections briefly discusses some potential senolytics and some recent findings. The information is summarised in Table 2.

#### 4.1.1. Dasatinib + Quercetin

Dasatinib + quercetin as a senolytic treatment was first identified by Zhu et al. using a hypothesis-based approach, by investigating drugs that interfere with the SCAPS, which were identified through transcriptomics [145,163]. On one hand, dasatinib is a known inhibitor of multiple tyrosine kinases and has also been found to eliminate senescent cells by disrupting the ephrin B (EFNB)-dependent suppression of apoptosis, one of the pathways by which senescent cells escape cell death [164,165,166]. Quercetin, on the other hand, is a natural flavanol with a range of bioactivities, such as anti-oxidation [167]. It was proposed that quercetin likely mediates apoptosis in senescent cells by inhibiting PI3K, which is involved in regulating several processes, including cell survival, and serpines (specifically plasminogen activator inhibitor), that have been found to protect tumour cells from apoptosis [163,168,169]. In a more recent study, quercetin was found to abolish the resistance of senescent IPF fibroblasts to death ligand-mediated apoptosis, by upregulating the death ligands Fas1 and Caveolin-1 which are responsible for a decreased sensitivity to pro-apoptotic stimuli in IPF, as well as in inhibiting Akt (a serine/threonine kinase involved in the PI3K/Akt pathway regulating cell survival) activation [170].

The dasatinib + quercetin combination (D+Q) has been extensively studied in mouse models for ageing and ageing related diseases, such as atherosclerosis [171]. Most recently, D+Q administration was found to eliminate senescent cells in the small intestine, alleviate intestinal inflammation, and modulate the microbiome in aged mice, suggesting a potential in improving gut health [150].

Given the numerous evidence regarding the effectiveness of D+Q in animal models, this combination has also progressed into several clinical trials. Currently, clinical trials are looking into D+Q in hematopoietic stem cell transplant survivors (NCT02652052), who are at risk for premature ageing, in diabetic chronic kidney disease (NCT02848131), and in Alzheimer’s disease (NCT04063124). In an initial report for the study into diabetic chronic kidney disease, a brief administration of D+Q in subjects showed success in the reduction of senescent cells in the adipose and skin tissue, as indicated by the decrease in the number of cells with the senescence markers p16, p21, and SA β-gal, a decrease in number of macrophages that were anchored in the adipose tissue by senescent cells, as well as the proliferation of adipocyte progenitors in primary culture that was derived from subjects’ biopsies [172].

However, a limitation of D+Q is that it may not be useful as a synergistic treatment with senescence-inducing chemotherapy. In Kovakovicova et al., the authors demonstrated that D+Q was not effective in clearing doxorubicin-induced senescent hepatocellular carcinoma cells [173].

#### 4.1.2. Navitoclax

Navitoclax is a BH3 mimetic drug which binds to the BH3 domain of pro-survival BCL-2 family proteins, and was found to be upregulated in senescent cells and identified as one of the anti-apoptotic pathways [83,163,174,175]. The blockade of the BCL-2 protein family/BH123 family protein interaction releases the pro-apoptotic BH123 proteins (BAK and BAX) for oligomerisation, thereby mediating the mitochondrial apoptosis pathway.

Navitoclax has been found to be effective in eliminating senescent cells in several radiation-induced senescent cell lines, such as human IMR90 fibroblasts and chondrocytes [175,176]. It has also been established in several mouse models, such as for senescent bone marrow hematopoietic stem cells, to promote their rejuvenation; for senescent white adipose tissue, to treat obesity-induced metabolic disorder; and for senescent type II alveolar epithelial cells (AECIIs), to potentially reverse persistent pulmonary fibrosis which is a side effect of thoracic radiation therapy [84,177,178]. In a recent study, navitoclax was found to eliminate cisplatin cytotoxic drug-induced senescent head and neck squamous carcinoma in vitro, but this result was not translated into in vivo mouse models in the same study [151].

However, the usage of navitoclax as a senolytic is challenging, as it has low selectivity and thus causes toxicity issues, including thrombocytopenia, as its target BCL-XL is important for platelet function. In a recent study, navitoclax was found to cause trabecular bone loss in aged mice, which was possibly due to its off-target effects on non-senescent BMSCs and osteoblasts [179]. To combat platelet toxicity, it has been proposed in a new study that the selectivity of navitoclax can be enhanced by galacto-conjugation, which allows navitoclax to be activated in the presence of SA β-gal in senescent cells [180].

Additionally, other BH3 mimetics with better selectivity have also been identified, including A1331852 and A1155463, which are BCL-XL specific inhibitors that appear to be less likely to cause neutropenia than navitoclax [181]. Venetoclax, a BCL-2 specific inhibitor with a better safety profile than navitoclax, was approved in 2016 for the treatment of chronic lymphatic leukaemia, suggesting the potential for safer future navitoclax-derived compounds [182].

#### 4.1.3. Fisetin

Fisetin is another natural flavonoid compound with a wide range of bioactivities, similar to quercetin, but with a reportedly higher potency [152]. Fisetin has been shown to be involved in many signalling pathways and in the promotion of apoptosis via the upregulation of pro-apoptotic BH123 proteins, the downregulation of NF-κB and the anti-apoptotic BCL-2 family proteins, and the modulation of the PI3K/Akt/mTOR pathway [183,184,185,186].

Fisetin has been found to be a potent senolytic in mouse models. The administration of Fisetin on aged wild-type mice reduced senescence markers in the fat, spleen, kidney, liver, and a subset of cell types in the adipose tissue, and its long-term administration on wild-type mice late in life improved tissue homeostasis, suppressed ageing-associated diseases like osteoporosis, and extended their median and maximum lifespan [152]. Fisetin has also been demonstrated to rescue bleomycin-induced pulmonary fibrosis in mice by relieving senescence in alveolar epithelial cells [153].

In terms of clinical studies, Fisetin is currently under investigation by the Steadman Philippon Research Institute for osteoarthritis in a Phase I/II randomised, double-blind study for clinical efficacy and safety, in which one of the markers measured will be whether Fisetin reduces senescence in joint health (NCT04210986).

An advantage of Fisetin is its low toxicity even at high dosages [152,181]. However, cell-specific senolytic activity is likely to be a barrier to its potential as a broad senolytic: Fisetin was found to induce apoptosis in senescent human umbilical vein endothelial cells (HUVECs), but not senescent IMR90 cells, a human lung fibroblast strain, nor primary human preadipocytes, suggesting a limited use of Fisetin in different contexts [181].

#### 4.1.4. HSP90 Inhibitors

Heat shock protein 90 (HSP90) is a ubiquitous chaperone protein that is critical in the ATP-dependent folding and stabilisation of proteins under physiological and stress conditions, and which plays many roles in cellular processes, such as apoptosis and cell cycle control [187]. It is suggested that HSP90 inhibition exerts senolytic effects by downregulating the anti-apoptotic PI3K/Akt signalling pathway—in a study by Fuhrmann-Stroissnigg et al. in which the first HSP90 inhibitors like Geldanamycin were first identified for having senolytic effects, this experiment found that HSP90 inhibitors block the activation phosphorylation of Akt, resulting in an increased susceptibility to apoptosis [154]. Most HSP90 inhibitors that have been identified exert their inhibitory effects by binding to the N-terminal ATP pocket of HSP90.

In the first study to identify senolytic HSP90 inhibitors, HSP90 inhibitor 17-DMAG (derivative of Geldanamycin) was found to reduce senescence in oxidative stress-subjected murine mesenchymal stem cells, etoposide-subjected human IMR90 cells, and telomere-shortened human WI38 cells (fibroblast). In the same study, in a DNA-repair-impaired ERCC1-deficient mouse model, HSP90 inhibitors improved health and reduced the senescence marker p16 expression by 50%, thereby establishing in vivo activity [154].

However, there have been varying levels of success with HSP90 inhibitors in clinical studies. Some were either not studied in clinical settings or were discontinued, due to reasons such as toxicity and an undesirable pharmacological profile. Currently, two HSP90 inhibitors, ganetespib and AT13387, are under active clinical studies. The clinical investigational status of the different HSP90 inhibitors was recently summarised in Gupta et al. [155].

Given that multiple isoforms of HSP90 are expressed in several cellular organelles (cytosol, mitochondria, endoplasmic reticulum, and sometimes the nucleus), it has been suggested that they can be used to target many cellular processes. However, to overcome the implicit complexity (for example, the enhanced expression of different isoforms in different cancers), organelle-specific HSP90 inhibitors have been proposed [188]. Another advantage of HSP90 inhibitors is that they have been shown to work in a variety of senescent cell types induced by different stresses, all while preferentially killing senescent cells over non-senescent cells [154].

However, it has been suggested that HSP90 inhibition may, conversely, also promote senescence. As HSP90 may play a role in suppressing senescence by downregulating ARF and stabilising telomerase reverse transcriptase enzymes, inhibiting HSP90 may lift the suppression of senescence [189,190]. It is likely that the pro-senescence and anti-senescence effects of HSP90 inhibitors are dependent on concentration and context.

#### 4.1.5. FOXO4 Inhibitors

FOXO4 proteins can promote senescence by upregulating the expression of p21 [191]. In irradiation-induced senescence, senescence is maintained via FOXO4 binding to p53 at the site of DNA damage (in PML), which prevents the nuclear export of p53 [192,193]. This, in turn, increases p21 expression, thus maintaining a p16-independent senescence response. FOXO4–DRI, developed by Baar et al., disrupts the FOXO4/p53 axis by competing with FOXO4 for p53 binding [193]. The binding of the inhibitor and p53 causes the nuclear exclusion of p53. This event leads to the interaction of p53 with BCL–XL, which subsequently causes cytochrome c release and thus results in mitochondrial-mediated apoptosis via caspase3/7 activation [194].

FOXO4-DRI has been shown to eliminate senescent chemotoxic(doxorubicin)-exposed human IMR and revitalise fast-ageing and naturally-ageing mice models (in terms of hair loss, fitness, and renal function) [193]. FOXO4–DRI selectively reduced viability in senescent human IMR90 when compared to non-senescent cells, with an 11-fold difference [193]. Most recently, FOXO4–DRI demonstrated effectiveness in eliminating FOXO4-assisted senescent mouse Leydig cells in vitro and in vivo, and improved the testicular tissue microenvironment by downregulating several SASP factors, such as cytokine IL-1α, which led to the improvement of age-related testosterone secretion insufficiency [156].

#### 4.1.6. Cardiac Glycosides

Cardiac glycosides are known inhibitors of the Na^+^/K^+^ ATPase, which maintains membrane potential by coupling the export of 3 Na^+^ to 2 K^+^. It is speculated that, at least in part, this mechanistic action contributes to the elimination of senescent cells. Triana-Martinez et al. demonstrated that digoxin causes the depolarisation of membrane potential in senescent A549 lung tumour cells. This change in the concentration of intracellular Na^+^ (high) causes the blockade of the Na^+^/H^+^ exchanger, thereby preventing the export of H^+^ and thus causing intracellular acidification, which drives the cell into apoptosis [157]. It was also suggested that the cardiac glycoside (oubain) may cause apoptosis by elevating the levels of pro-apoptotic BCL-2 family proteins, namely NOXA [158].

Triana-Martinez et al. had demonstrated that digoxin alleviates senescence in A549 cells which were exposed to a wide variety of chemotherapeutic agents, such as bleomycin and doxorubicin, and this effect translated to a robust antitumour response in mouse models treated with digoxin and the chemotherapeutic drug gemcitabine, suggesting its potential as a complement to chemotherapy [157]. Oubain was also shown to be a broad-acting senolytic in Guerro et al. that, on top of acting on senescent preneoplastic cells and senescent cells induced by chemotherapy, oubain also acted on age-associated senescent cells and reduced the local inflammation and immune infiltration in old mice [158]. Additionally, cardiac glycosides have also been shown to reduce atherosclerosis and bleomycin-induced pulmonary fibrosis in mice [159,160].

The effective concentration of cardiac glycosides for senolytic activity may depend on cell type as well as context, and may fall within or exceed a safe plasma concentration; the studies have been contradictory. For example, Guerrero et al. reported that digoxin exerts effects at concentrations which are close to those observed in the plasma of patients treated with the drug, while the digoxin optimal concentration is reportedly 200-fold higher than the safe plasma concentration [158,195]. Although digoxin’s toxicity is well-documented and tolerated in the treatment for cardiac failures, there needs to be a risk–benefit analysis if it were to be used for less imminent problems such as ageing-related treatments.

#### 4.1.7. Proteolysis Targeting Chimeras (PROTACs)

Proteolysis-targeting chimeras (PROTACs) refer less to a specific molecule but more to a technology or strategy for treatment to improve the profiles of drug action. PROTACs are bifunctional, small molecules which consist of three main components: a ligand that is recognised by the target protein, a ligand that recruits a specific E3 ligase, and a linker that connects the two ligands [196]. The PROTACs engage the ubiquitin proteasome system (UPS)—the PROTACs bring E3 ligases in close proximity to target proteins so as to initiate the ubiquitination of the target, so that it can subsequently be tagged for proteasomal degradation. In this way, PROTACs can improve the selectivity of senolytic drugs, which would lower the toxicity of these drugs, a huge problem that is present in current treatment. Thus, PROTAC presents a huge potential for senolytic treatment.

The first time the PROTAC technology was applied to a senolytic drug was on navitoclax. In He et al., the researchers attempted to reduce the toxicity of navitoclax to platelets by converting it into PZ15227 (PZ), a BCL–XL-specific molecule which consists of a component of navitoclax which binds to BCL–XL, conjugated to E3 ligase cereblon (CRBN), which is poorly expressed in platelets but abundantly in other cells [161]. Consequently, PZ can target pro-survival BCL–XL for degradation, and mediate apoptosis in senescent cells but insignificantly in platelets, thus preventing thrombocytopenia. Indeed, the study found that PZ affected platelets and non-senescent cells (as they do not upregulate BCL–XL for survival) minimally, but potently in ionising radiation-induced senescent WI38 cells, and this activity was 37 times less toxic than navitoclax. This success was also replicated in extensive replication-induced senescent WI38 cells, as well as irradiation-induced senescent renal epithelial and pre-adipocyte cells, suggesting a broad spectrum of activity. More importantly, this result translated to an in vivo mouse model: PZ was found to improve osteoprogenitor function and rejuvenate hematopoietic stem cells by clearance of senescent cells following its administration, without inducing severe thrombocytopenia.

The sub-stoichiometric catalytic activity of PROTAC technology results in PZ’s improved potency and toxicity when compared to navitoclax, and implies that PZ is potentially a better drug candidate, as administration may only require a low concentration and exposure [161]. This potency is circumstantial, however, as different senescence employs different SCAPs: PZ’s activity was not found to be different from navitoclax in some cell types, such as irradiation-induced senescent IMR90 cells. Another limitation of the current PZ is that it may not be totally without platelet toxicity, since PZ binds with high affinity to BCL–XL and may still inhibit the pro-survival BCL-2 family protein. For this, the researchers proposed strategies such as using E3 ligases that are minimally expressed in platelets, such as the von Hippe-Lindau.

Another study which identified the BET family protein degrader as a potential novel senolytic agent experimented with a PROTAC, ARV825 (which tags the BRD4 for ubiquitination by CRBN), with much success, in which the treatment eliminated senescent hepatic stellate cells in obese mouse livers and chemotherapy-induced senescent cells in an immunocompromised mouse model with xenografted tumours [162]. The authors attributed this senolytic activity, in part, to two independent pathways: first, the blocking of non-homologous end joining repair, which promotes double-stranded DNA breaks and secondly, the upregulation of genes involved in autophagy. Together, these experiments suggest that the PROTAC technology is indeed very promising in enhancing senolytics in terms of their pharmacological profile, which is an exceedingly important aspect of drug development.

Another branch of senotherapy, termed “senomorphics”, deals with the attenuation of the SASP, which impedes the progression of dysregulated senescence, rather than the induction of apoptosis in senescent cells. Under this category, rapamycin and metformin are amongst the more reputable drugs. A potent mTOR inhibitor, rapamycin has been shown to impede senescence and increase lifespan in mouse models [197,198,199]. By inhibiting mTOR, an important kinase molecule regulating numerous metabolic processes, such as protein synthesis, rapamycin is thought to hamper protein synthesis and subsequently make the highly active senescent phenotype inviable, as well as suppress the SASP. which diminishes its ability to exert a paracrine influence and to induce pathologies in neighbouring cells [200,201,202]. In Laberge et al., the authors proposed that rapamycin selectively reduced the translation of IL1A, a cytokine upstream of transcriptional factor NF-κB, which, in turn, diminished the NF-κB-controlled mRNA levels of the SASP, including inflammatory cytokines such as IL6. As a result of this attenuated SASP, the ability of senescent fibroblasts to induce prostate tumours in mice were found to have declined. However, the inhibition of mTOR has been observed to cause multiple side effects, including thrombocytopenia, as mTOR is a crucial signalling molecule in various pathways [203,204] and it has been alleged that studies thus far on rapamycin’s potential as a senomorphic have been carried out at sub-optimal doses. Therefore, this may present difficulties in translating laboratory experiments into the meaningful clinical use of rapamycin. Similarly, metformin, a prescribed anti-diabetic drug, has also been demonstrated to prevent senescence formation and is thought to do so by attenuating the SASP via interfering with the NF-κB pathway as well [205,206]. Moiseeva et al. found that Metformin curbed the SASP expression through the deactivation of IKB kinase, another upstream activator of NF-κB [207]. More recently, an investigation by Lim et al. uncovered a novel senomorphic drug, avenanthramide C (Avn C) [208]. Avn C was found to alleviate a few senescent markers, including SA β-gal, in liposaccharide-induced and inflammation-induced senescent human diploid fibroblasts. It was posited that Avn C interferes with the SASP expression by activating the AMPK signalling pathway, which subsequently hampers the p38/NF-κB signalling pathway.

However, it seems that no senotherapeutic drug works universally on different senescent cells [209]. These drugs may work in certain cell types but not others; and even so, within the same cell type, they may not work on different strains. For example, navitoclax, which targets the BCL-2 family SCAPs, has shown to be senolytic in cultured human umbilical vein endothelial cells but not in primary fat cell progenitors, as well as having senolytic activity in cultured lung fibroblast-like cells but not in primary lung fibroblast-like cells [175].

Despite having some limitations, senotherapeutic drugs do present a couple of advantages that address concerns of modern medicine. Due to the mechanisms that maintain the senescence programme being so sensitive, transient disruption in the SCAPs can be very effective in clearing senescent cells. This means that senolytic drugs need not be administered continuously for a long period of time [163,209]. This acute form of treatment makes the implementation of senolytic treatment highly feasible, and could possibly prevent side effects from developing, as well as prevent any potential off-target activity [209]. How regular the administration will then need to be will depend on the rate of re-accumulation of senescent cells, which is likely to differ between individuals. In a framework which was proposed to explain senescent cell accumulation based on turnover rate, the senescent cell level in 3 months old mice were found to have a half-life of 4.7 days when compared to 18 days in 22 months old mice [210]. It is suggested that the balance between the production and clearance of senescent cells in young mice are more effectively maintained, while, in older mice, production is heightened (increasing linearly with age) and removal rate (which can also be affected by the context) is slowed, thus resulting in large variations in senescent cell accumulation at old age. Additionally, another possible advantage of senolytics is that cell division-dependent drug resistance may not become an issue. Cell division dependent drug resistance is acquired when mutations occur during the replication process which confers daughter cells the ability to withstand drugs through various mechanisms, and this allows the propagation of future generations [211]. Senescent cells, by definition, do not have the ability to divide, and hence cannot acquire such mutations that can diminish the effectiveness of the drugs [3,209].

Although ageing is a natural biological phenomenon that is observed across different divisions of life (animals, plants and even fungi), there are organisms which are considered to be exceptions and believed to show “negligible senescence” [212]. These organisms, such as the Rougheye rockfish, which has an average lifespan of 205 years, seemingly do not show any functional decline or increased mortality rates with age [212,213]. The Strategies for Engineered Negligible Senescence (SENS) is a term coined by the biogerontologist Aubrey de Grey, and frames a field of regenerative medical therapy whose aim is allow us attain this state of “negligible senescence” [214]. Although the SENS framework is highly controversial and dismissed by many as science fiction, it is undeniable that senotherapy remains one of the most promising avenues in our pursuit of longevity. Case in point, a recent study by Johmura et al. discovered senolytic potential in glutaminase inhibitors, a class of drug molecules currently under study for anti-cancer therapy. Glutaminase is a mitochondrial enzyme that plays a variety of roles such as the regulation of acid balance [215]. In this study, Johmura et al. demonstrated the importance of glutaminase in a senescent human fibroblast cell line (hHCA2) which was induced through different methods, such as nutlin3a and oxidative stress, in maintaining the viability of these cells, and inhibition of glutaminase led to the death of the senescent cells by preventing the neutralisation of intracellular acid build-up, caused by the leakage of lysosomal H^+^ ions. When administered in a mouse model, the glutaminase inhibitor ameliorated several ageing-related diseases such as glomerulosclerosis, atherosclerosis, lung fibrosis, and kidney dysfunction. Although the clinical development of senolytics is in its infancy stage, the belief in senolytic drugs is so strong that it has motivated a whole community which is led by self-experimentation with these drugs.

### 4.2. Pro-Senescence Therapy

Pro-senescence therapy deals mainly with the elimination of cancer, through the induction of senescence in tumourigenic cells to stop the proliferation of these rogue cells which interfere with tissue functioning. For example, CDK 4/6 inhibitors are amongst the most promising pro-senescence agents, which act through reducing the phosphorylation of Rb proteins, which prevents the activation of E2F transcription factors and, subsequently, block the progression into the S phase of the cell cycle, and these have been approved to treat metastatic breast cancer [216]. The treatments using CDK 4/6 inhibitors are associated with the implementation of the SASP, as studies have found that the SASP can engage the immune system in various ways, including through the recruitment of cytotoxic T-cells in breast cancer models, or NK cells in lung cancer, as well as the suppression of Tregs [217,218,219]. Another potential novel pro-senescence strategy that has been identified of late is the inhibition of isocitrate dehydrogenase 1 (IDH1) for high-grade serous cancer (HGSC) in epithelial ovarian cells [220]. As part of metabolic reprogramming, in the establishment of the cancer programme, HGSC cells have been found to upregulate the TCA cycle and the IDH1 gene, which HGSC cells use to convert glucose to α-ketoglutarate. The study found that knockdown or inhibition of this gene in an ovarian cancer cell line induced senescence in these cells, which was indicated by an increased SA β-gal activity, decreased lamin B1, and increased PML bodies observed. It was suggested that the induction into senescence was due to the inhibition of α-ketoglutarate production, which then increased the activity of transcription-repressive histone H3K9 demethylase at multiple E2F target genes, leading to cell arrest [221,222]. Moreover, the authors found that the SASP was not as significantly upregulated in IDH1 inhibition-induced senescence when compared to etoposide-induced senescent cells, further suggesting that this novel approach may be without the detrimental effects of TIS.

Another interesting mechanism in which pro-senescence therapy can lead to cancer elimination was explored recently by Ruscetti et al. [223] In a mouse model of pancreatic cancer, the induction of senescence with CDK4/6 inhibitors resulted in a pro-angiogenesis factor-containing SASP that led to tumour vascularisation. This vascular remodelling was shown to promote the uptake and action of the chemotherapeutic drug gemcitabine in the tumour, as well as enhanced the filtration of CD8^+^ cells. Again, this evidence suggests that pro-senescence could potentially add value to current treatment strategies (which is to induce cell death directly), since senescent cells have non-cell autonomous signalling effects.

As previously mentioned, the SASP plays an important role in the tissue microenvironment, and can either suppress tumourigenesis or promote tumour formation through paracrine signalling [5,35]. Because the SASP is so potent, there have been further strategies proposed to complement pro-senescence treatment: to reprogramme the SASP so as to attenuate its catastrophic effects [224,225]. In a study probing into the senescence escape of TIS tumour cells, it was found that senescent non-small-cell lung cancer (H460 cell line) mice showed a slower growth in the immunocompetent mouse model when compared to the immunocompromised mouse model, suggesting that the immune system acts upon senescent cells to suppress their tumour-inducing capacity [131]. In another instance, the inhibition (both pharmacologically and genetically) of Janus kinase 2 (JAK2), a signal transducer and an activator of the transcription 3 (STAT3) pathway, in PTEN-loss-induced cellular senescent (PICS) tumours in mice was found to enhance chemotherapy, by decreasing the level of immunosuppressive cytokines while maintaining the level of immunostimulatory cytokines, which allowed for the induction of a strong anti-tumour immune response [225]. All of this evidence represents a potential avenue for exploiting senescence surveillance in anti-cancer treatment, which could improve therapeutic efficacies.

While pro-senescence therapy is a promising strategy, the manipulation of the senescence programme in therapeutic applications is delicate [224]. The SASP is a complex array, and the clearance of senescent cells by engaging the immune system, or complementing the treatment with senolytics, is imperative to prevent the development of detrimental effects [195]. In addition, the lack of ease in identifying senescent cells, as a result of the absence of universal biomarkers, also further complicates the assessment of senescence in evaluating the effectiveness of senescence therapy, thus impeding the development of pro-senescence programmes [226].

## 5. Conclusions

The progress in the development of senescence-associated therapy has been a roller-coaster ride. The results of the pilot human clinical trial that tested senolytic drugs for feasibility, a study released earlier in the year 2019, marked a milestone in senolytic research and likely reinvigorated the passion for senescence studies in the scientific community; yet, a recent Unity Phase 2 clinical trial of a candidate drug, UBX0101, to treat osteoarthritis has shown disappointing results, despite being only halfway through its intended period of study. Although the research on senescence is in its early stages and still mainly relies on a hypothesis-driven approach, it is hopeful that as more is learned about senescent cells, the wealthier is the knowledge that can be applied onto these therapeutic studies [149]. Moving forward, there remains much dedicated work to be done. The discovery of biomarkers, both universal or specific, that characterise senescent cells is still a troubling difficulty; the understanding of the complex factors of senescence is still shallow, and senescence-targeted therapies are still steps too far from clinical use. However, it is true that with the continual advancements in biotechnology and the biological sciences, senescence will soon be more meaningful than just the beginning of a cell’s end. Although senescence plays a complex role in the pathology of cancer and ageing-related disease, including neurodegenerative diseases such as Alzheimer’s, this means that there exists numerous ways to manipulate the senescence programme—be it through inducing senescence in tumour cells, eliminating and preventing senescence of dysfunctional endothelial cells which are predisposed to atherosclerosis, or the precise alteration of the SASP—supporting senescence’s role as an attractive and potential target for both chronic and terminal diseases. Considering the accelerating development of biologics, particularly antibodies, perhaps future therapeutic studies on senescence might focus on this bracket of therapeutic drugs which comes with some advantages, such as the reduction of off-target toxicity.

## Figures and Tables

**Table 1:** Classical SASP factors and examples.

SASP Factors	Examples	Effects and Mechanisms
Cytokine	IL-6	- Enforces senescence via activation of STAT3 [44] - Immunosuppression via increase in myeloid-derived suppressor cells [45] - Anti-tumorigenesis via recruitment of macrophages and natural killer T cells [46,47]
Growth Factors	TGF- β1	- Enforces senescence via ROS-mediated DDR; via miR-29a/c upregulation [48]
Proteases	MMPs	- Plaque instability via fibrous cap weakening [49]
Non-Protein Factors	miRNA	- Immunomodulation via downregulation of cytokine IL-6 & IL-8 [50]

**Table 2:** Summary of major and upcoming senolytic drugs, their status of investigation and recent updates.

Senolytic Drugs	Mechanisms	Recent Updates
Dasatinib + Quercetin (D+Q)	Promoting apoptosis by disrupting multiple SCAPs including ephrin-dependent suppression of apoptosis, PI3K/Akt signalling as well as by upregulating Fas-1 and Caveolin-1.	Improving gut health [150]. Clinical trials in Hematopoietic Stem Cell Transplant Survivors (NCT02652052) who are at risk for premature ageing, in diabetic chronic kidney disease (NCT02848131) and Alzheimer’s (NCT04063124).
Navitoclax	Interfering with BCL-2 protein family/BH123 protein family interaction and mediates mitochondria-dependent apoptosis.	Elimination of cisplatin cytotoxic drug-induced senescent head and neck squamous carcinoma in vitro [151].
Fisetin	Promoting apoptosis via SCAPs interference: upregulate pro-apoptotic BH123 proteins, downregulation of NF-κB and anti-apoptotic BCL-2 family proteins and modulation of the PI3K/Akt/mTOR pathway.	Health span and lifespan improvement in mice model [152]. Improvement of bleomycin-induced pulmonary fibrosis condition in mice by relieving senescence in alveolar epithelial cells [153]. Clinical study by the Steadman Philippon Research Institute for osteoarthritis (NCT04210986).
HSP90 inhibitor	Downregulating the anti-apoptotic PI3K/Akt signalling pathway.	Reduced senescence in multiple cell models including telomere-shortened human fibroblast cell and improved health span in mice model [154]. Clinical investigation of ganetespib and AT13387 [155].
FOXO4 inhibitor	Disrupting the FOXO4/p53 axis by competing with FOXO4 for p53 binding, leading to the nuclear exclusion of p53 and its interaction with BCL-XL which subsequently causes cytochrome c release and thus causing mitochondrial-mediated apoptosis via caspase3/7 activation.	Elimination of FOXO4-assisted senescent mice Leydig cells in vitro and in vivo [156].
Cardiac Glycoside	Inhibiting Na^+^/K^+^ ATPase which causes the blockade of the NA^+^/H^+^ exchanger, thus preventing the export of H^+^ which results in intracellular acidification and ultimately, apoptosis. Additionally, may also cause apoptosis by elevating levels of pro-apoptotic BCL-2 family proteins.	Alleviating senescence in A549 cells exposed to a wide variety of chemotherapeutic agents [157]. Alleviating age-associated senescent cells and reduced local inflammation and immune infiltration in old mice [158]. Improving atherosclerosis and bleomycin-induced pulmonary fibrosis in mice [159,160].
PROTAC	Technology which improves the pharmacological profiles of senolytic drugs. PROTACs consists of 3 components: a ligand that recognises the target, a ligand that recognises an E3 enzyme and a linker that connects the two ligands.	Navitoclax-derived PROTAC: PZ15227 [161]. BET family protein degrader-derived PROTAC: ARV825 [162].

## Data Availability

Not applicable.
